# Intensity-modulated radiation therapy versus three-dimensional conformal radiation therapy with concurrent nedaplatin-based chemotherapy after radical hysterectomy for uterine cervical cancer: comparison of outcomes, complications, and dose-volume histogram parameters

**DOI:** 10.1186/s13014-015-0486-5

**Published:** 2015-08-25

**Authors:** Fumiaki Isohashi, Seiji Mabuchi, Yasuo Yoshioka, Yuji Seo, Osamu Suzuki, Keisuke Tamari, Michiko Yamashita, Hikari Unno, Yasuto Kinose, Katsumi Kozasa, Iori Sumida, Yuki Otani, Tadashi Kimura, Kazuhiko Ogawa

**Affiliations:** Department of Radiation Oncology, Osaka University Graduate School of Medicine, 2-2 (D10) Yamadaoka, Suita, Osaka 565-0871 Japan; Department of Obstetrics and Gynecology, Osaka University Graduate School of Medicine, Suita, Osaka Japan

## Abstract

**Background:**

The purpose of this study is to report our clinical outcomes using intensity-modulated radiation therapy (IMRT) for adjuvant treatment of cervical cancer, compared with three-dimensional conformal radiation therapy (3DCRT), in terms of tumor control, complications and dose-volume histogram (DVH) parameters.

**Methods:**

Between March 2008 and February 2014, 62 patients were treated with concurrent nedaplatin-based chemotherapy and whole-pelvic external beam radiation therapy (RT). Of these patients, 32 (52 %) received 3DCRT and 30 (48 %) received IMRT.

**Results:**

The median follow-up periods were 40 months (range 2–74 months). The 3-year overall survival rate (OS), locoregional control rate (LRC) and progression-free survival rate (PFS) were 92, 95 and 92 % in the IMRT group, and 85, 82 and 70 % in the 3DCRT group, respectively. A comparison of OS, LRC and PFS showed no significant differences between IMRT and 3DCRT. The 3-year cumulative incidences of grade 2 or higher chronic gastrointestinal (GI) complications were significantly lower with IMRT compared to 3DCRT (3 % vs. 45 %, *p* < .02) and in patients with V40 of the small bowel loops of ≤340 mL compared to those with >340 mL (3 % vs. 45 %, *p* < .001). Patients treated with IMRT had a higher incidence of grade 3 acute hematologic complications (*p* < .05). V40 and V45 of the small bowel loops or bowel bag were predictive for development of both acute and chronic GI complications.

**Conclusions:**

Our results suggest that IMRT for adjuvant treatment of cervical cancer is useful for decreasing GI complications without worsening outcomes.

## Background

Adjuvant whole-pelvic radiation therapy (RT) concurrent with platinum-based chemotherapy is standard treatment for patients after radical hysterectomy for uterine cervical cancer with high-risk clinicopathological factors [1]. For patients with intermediate-risk factors, whole-pelvic RT with or without chemotherapy can at least reduce locoregional recurrence [2, 3]. However, patients undergoing whole-pelvic RT with or without chemotherapy after radical hysterectomy may suffer acute and chronic gastrointestinal (GI) complications.

We previously reported that dose-volume histogram (DVH) parameters of the small bowel loops were predictive for development of chronic GI complications and that V40 of the small bowel loops >340 mL was an independent risk factor for chronic GI complications using conventional two-dimensional (2D) or three-dimensional (3D) conformal RT (CRT) concurrently with nedaplatin [4]. There is often a significant amount of small bowel in the pelvis that can be avoided to a greater degree with intensity-modulated RT (IMRT) than with 3DCRT. Therefore, since October 2010, we have used IMRT as adjuvant whole-pelvic RT concurrently with nedaplatin. The purpose of this study is to report our clinical outcomes using IMRT for adjuvant treatment of cervical cancer, compared with 3DCRT, in terms of tumor control and complications. We also evaluated whether DVH predictors for development of GI complications using 2D or 3DCRT were also useful parameters in IMRT.

## Methods

### Patients

The study was performed as a retrospective chart review and was approved by our institutional review board. A total of 102 patients with clinical stage IB1-IIB uterine cervical cancer underwent radical hysterectomy and postoperative RT at our institute between March 2008, when we changed from 2D to 3DCRT in postoperative concurrent nedaplatin-based chemoradiation therapy, and February 2014. Postoperative RT is indicated when a patient’s pathological report displays any one of the following high-risk prognostic factors: parametrial invasion, pelvic lymph node metastasis, a positive surgical margin, or one of the following intermediate-risk prognostic factors: deep stromal invasion, lymphovascular invasion, or a large tumor (>4 cm in diameter) [5, 6]. Forty patients were excluded from the study: 12 who received extended-field RT alone because of multiple lymph node metastases [7], 12 who underwent clinical trials of whole-pelvic RT with concurrent carboplatin and paclitaxel [8], 13 who refused concurrent chemotherapy, and 3 who received intracavitary brachytherapy because of a close surgical margin. Thus, data were retrospectively analyzed for 62 patients treated with concurrent nedaplatin-based chemotherapy and whole-pelvic external RT.

### Radiotherapy and chemotherapy

Whole-pelvic RT was delivered with 3DCRT planning in 32 patients between April 2008 and September 2010, and with IMRT planning in 30 patients starting in October 2010. Whole-pelvic RT with 3DCRT or IMRT was performed as previously described [4, 8]. The differences between 3DCRT and IMRT planning are summarized in Table 1. The clinical target volume (CTV) was defined according to the consensus guidelines of the Radiation Therapy Oncology Group (RTOG) 0418 [9] and the atlas on the RTOG site, or using the Japanese Clinical Oncology Group (JCOG) guidelines [10]. The RTOG guidelines include a central vaginal CTV (proximal vagina and paravaginal tissues) and a CTV for the pelvic lymph nodes, whereas the JCOG guidelines include only a CTV for the pelvic nodes. We started using 3DCRT in 2008, and CTVs (central vaginal CTV and pelvic lymph nodes) were contoured using RTOG guidelines. In October 2010, we started to use IMRT, with pelvic lymph nodes contoured using JCOG guidelines and central vaginal CTV contoured using RTOG guidelines. Thus, in brief, CTVs in 3DCRT were contoured using RTOG guidelines and CTVs in IMRT were contoured using RTOG and JCOG guidelines.

**Table 1:** Summary of radiation-planning differences between 3DCRT and IMRT

		3DCRT	IMRT
Planning	slice	2.5 mm with normal quiet breathing	
CT	range	upper edge of L3 to at least 7 cm below the bottom of the obturator foramen	
	frequency	once (full bladder)	twice (full bladder and empty bladder)
RTP		XiO (Elekta, Stockholm, Sweden)	
CTV	regional nodal CTV	common iliac, external iliac, internal iliac and presacral	
	central vaginal CTV	proximal vagina and paravaginal tisusue	
PTV		1.0-cm uniform expansion of CTV	central vaginal CTV fused on both the full bladder and 0.7-cm uniform expansion of CTV
Dose	total (Gy)	50	50.4
	fractions	25	28
	prescri ption	center of the PTV	mean dose to the PTV
Normal structure	delineate before treatment	-	bladder, rectum, bowel bag and femoral head
	delineate after treatment	bowel bag, small bowel loops, large bowel loop, pelvic bone	small bowel loops

*3DCRT* three-dimensional conformal radiation therapy, *IMRT* intensity-modulated radiation therapy, *CT* computed tomography, *CTV* clinical target volume, *PTV* planning target volume

During the 3DCRT era, no normal structures were contoured before treatment. In IMRT planning, the bladder, rectum, bowel bag and femoral head were contoured before treatment because of the use of normal tissue constraints. The bowel bag for 3DCRT and the small bowel loops, large bowel loop and pelvic bone for 3DCRT and IMRT were contoured retrospectively for analysis in this study. The contouring methods for the bowel bag, small bowel loops and large bowel loop have been previously described [4]. The pelvic bone was contoured as described by Mell et al. [11].

In IMRT, target criteria and normal tissue constraints have been previously described [8]. The pelvic bone was not included as a planning constraint.

Nedaplatin (40 mg/m^2^) was given intravenously on a weekly basis for 5–6 weeks during the course of whole-pelvic RT, as previously described [4, 5].

### Evaluation of complications

GI, genitourinary (GU), and hematologic (HT) complications were assessed according to the Common Terminology Criteria for Adverse Events version 4.0. All patients received treatment with hospitalization. For acute complications, the patients were assessed for toxicity directly during treatment on a daily basis for GI and GU complications and on a weekly basis for HT complications. Thus, acute toxicity data including grade were collected prospectively. However, for chronic complications, toxicity data including the grade of each complication were collected retrospectively from follow-up records.

### Statistical analysis

Differences in clinicopathological factors, DVH parameters and incidence of complications between 3DCRT and IMRT were analyzed by Mann–Whitney *U* test for quantitative variables and by Fisher exact test for categorical variables. The actuarial overall survival rate (OS), loco-regional control rate (LRC) and progression-free survival rate (PFS) or incidence of chronic GI complications were calculated using the Kaplan-Meier method and differences between groups were compared by log-rank test. Correlations between grades of complications and DVH parameters were analyzed by analysis of variance (ANOVA). All statistical tests were two-sided and *p* < .05 or a 95 % confidence interval (CI) not encompassing 1 was considered significant.

## Results

The median follow-up periods from the start of RT were 40 months (range 2–74 months) for all patients, 57 months (5–74 months) for the 3DCRT group, and 28 months (2–44 months) for the IMRT group. Clinicopathological characteristics of the 3DCRT and IMRT groups are shown in Table 2. The characteristics were similar in the two groups, but the 3DCRT group had significantly more pathological T2 stage cases (44 % vs. 20 %, *p* = .04) and more pathological N1 stage cases (31 % vs. 20 %, not significant).

**Table 2:** Clinicopathological characteristics of patients treated with 3DCRT and IMRT

	3DCRT (*n* = 32)		IMRT (*n* = 30)		
	Median	Range	Median	Range	*p*
Age (y)	47	31-70	44	24-65	N.S.
BMI (kg/m^2^)	20.8	14.2-27.7	21.2	15.7-32.5	N.S.
Total nedaplatin (mg)	290	120-350	283	56-420	N.S.
	n	%	n	%	
Smoker	8	25	12	40	N.S.
T-stage					
T1	18	56	24	80	0.046
T2	14	44	6	20	
N-stage					
N0	22	69	24	80	N.S.
N1	10	31	6	20	
Histology					
SCC	23	72	16	53	N.S.
non-SCC	9	28	14	47	
DSI	31	97	29	97	N.S.
LVI	13	41	9	30	N.S.

*3DCRT* three-dimensional conformal radiation therapy, *IMRT* intensity- modulated radiation therapy, *BMI* body mass inex, *SCC* squamous cell carcinoma, *DSI* deep stromal invasion, *LVI* lymphovascular invasion

**Fig. 1:**
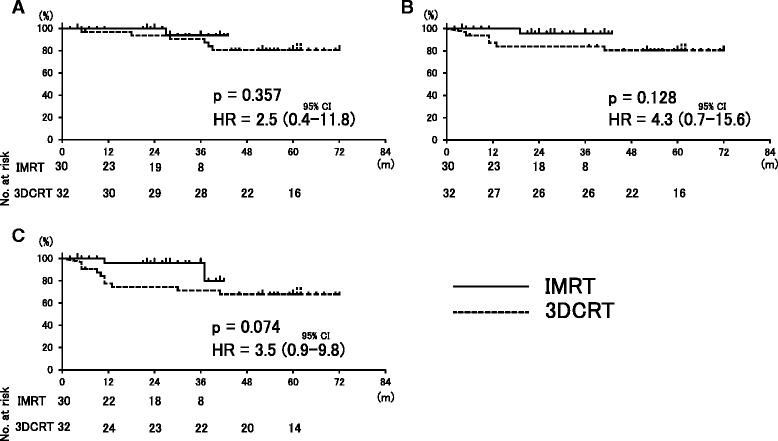
Kaplan-Meier estimates for comparison of **a** overall survival (OS), **b** locoregional control rate (LRC), and **c** progression-free survival (PFS) between IMRT and 3DCRT. OS, LRC and PFS did not differ significantly between the cohorts

The mean V95% values for the planning target volume were 97 % (range 91–99 %) and 97 % (93-100 %) in the 3DCRT and IMRT groups, respectively, with no significant difference between the groups (*p* = .32). Similarly, the mean V93% did not differ significantly between the groups (99 % vs. 99 %, *p* = .57). A comparison of OS, LRC and PFS also showed no significant differences between IMRT and 3DCRT (Fig. 1). The 3-year OS, LRC and PFS were 92 %, 95 % and 92 % in the IMRT group, and 85 %, 82 % and 70 % in the 3DCRT group, respectively.

**Fig. 2:**
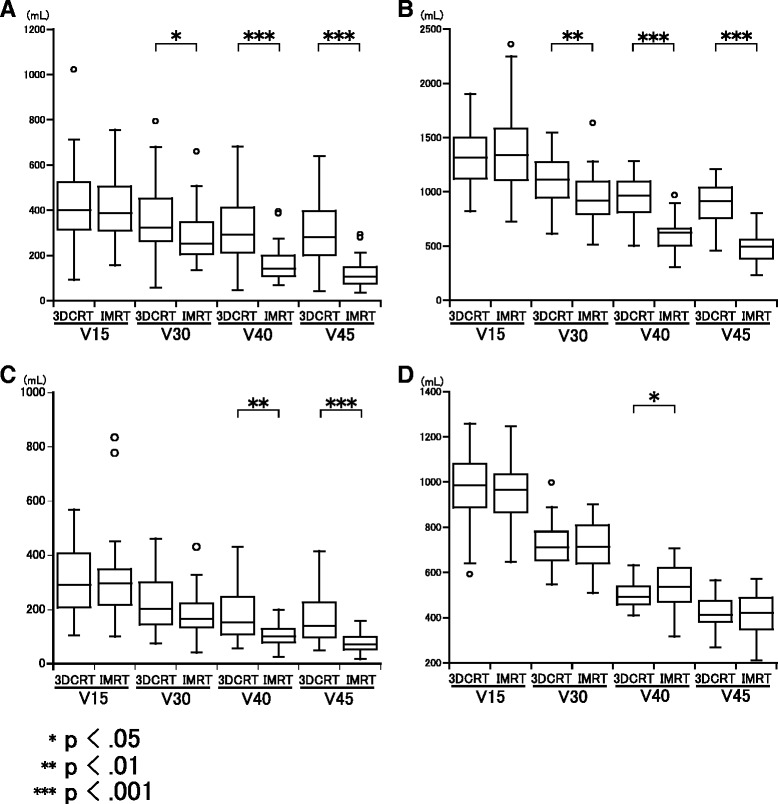
Comparison of DVH parameters for **a** small bowel loops, **b** bowel bag, **c** large bowel loop, and **d** pelvic bone between IMRT and 3DCRT

Comparisons of DVH parameters for small bowel loops, bowel bag, large bowel loop and pelvic bone between 3DCRT and IMRT are shown in Fig. 2, Tables 3 and 4. Patients who received IMRT had significantly reduced V40 and V45 volumes of the small bowel loops, bowel bag and large bowel loop, compared to patients who received 3DCRT (Fig. 2 and Table 3). Patients who received IMRT also had a reduced V30 of the small bowel loops and bowel bag, but a significantly increased V40 of the pelvic bone, compared to those treated with 3DCRT (Fig. 2 and Table 4).

**Table 3:** Comparison of DVH parametyers of bowels between 3DCRT and IMRT

		3DCRT				IMRT				
		Mean (cc)	Median (cc)	SE	range	Mean (cc)	Median (cc)	SE	range	*p*
Small bowel loops	V15	428	402	33	92-1021	411	386	27	156-755	0.688
	V30	362	325	28	58-793	289	253	22	136-660	0.018
	V40	316	293	26	48-683	164	143	15	69-395	<0.001
	V45	299	281	12	42-639	121	106	12	36-293	<0.001
	V15	1324	1314	50	821-1904	1347	1349	71	727-2360	0.972
	V30	1100	1112	41	615-1546	945	920	43	515-1635	0.001
Bowel bag	V40	944	968	36	505-1285	613	623	26	308-970	<0.001
	V45	891	917	35	459-1207	489	496	22	233-803	<0.001
Large bowel loop	V15	310	292	18	105-569	316	297	29	101-834	0.856
	V30	229	202	18	75-461	181	165	14	42-431	0.058
	V40	175	152	16	57-431	105	102	8	26-200	0.001
	V45	163	140	15	18-415	77	71	6	18-158	<0.001

*DVH* dose-volume histogram, *3DCRT* three-dimensional conformal radiation therapy, *IMRT* intensity- modulated radiation therapy, *SE* standard error, *V15-V45* volume receiving more than respective dose

**Table 4:** Comparison of mean DVH parameters of pelvic bone between 3DCRT and IMRT

	3DCRT			IMRT			
	Mean	SD	SE	Mean	SD	SE	*p*
(%)							
V10	90.5	9.0	1.6	92.3	2.8	0.5	0.724
V15	87.9	8.9	1.6	88.2	3.0	0.6	0.054
V20	85.7	8.8	1.5	80.4	4.2	0.8	<0.001
V30	65.2	6.1	1.1	66.2	4.4	0.8	0.341
V40	45.6	6.0	1.1	50.0	6.5	1.2	0.003
V45	38.7	6.8	1.2	38.1	7.3	1.3	0.827
(cc)							
V10	1001	154	27	1008	128	12	0.983
V15	972	149	26	965	130	24	0.568
V20	947	145	26	879	128	23	0.019
V30	719	98	17	721	103	19	0.827
V40	499	54	10	544	96	18	0.042
V45	424	65	11	415	94	17	0.849

*DVH* dose-volume histogram, *3DCRT* three-dimensional conformal radiation therapy, *IMRT* intensity-modulated radiation therapy, *SD* standard deviation, *SE* standard error, *V10-45* volume receiving more than respective dose

**Fig. 3:**
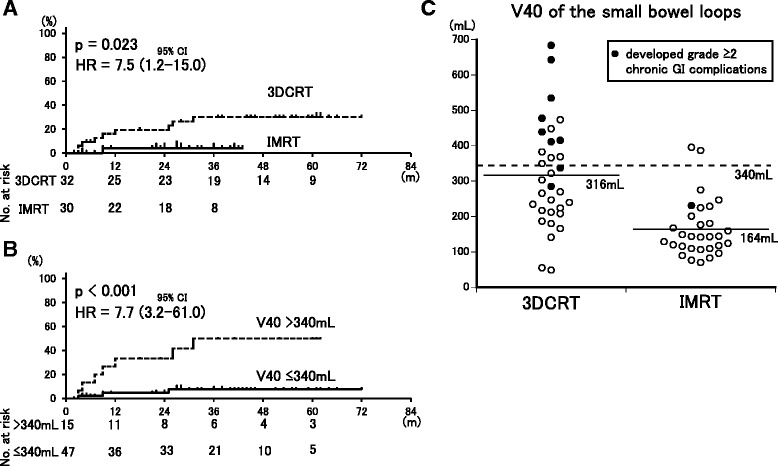
Kaplan-Meier estimates of cumulative incidence curves for **a** grade 2 or higher chronic GI complications between IMRT and 3DCRT, and **b** stratified by V40 of the small bowel loops. **a** Patients who received IMRT had fewer chronic GI toxicities than those treated with 3DCRT. **b**, **c** Patients with V40 > 340 mL had higher rates of grade 2 or higher GI complications compared to those with V40 ≤ 340 mL

The grades of acute or chronic complications and numbers of patients with these complications are summarized in Table 5. IMRT patients had fewer acute and chronic GI complications than those treated with 3DCRT, with the IMRT group having significantly fewer grade 2 or higher acute GI complications (63 % vs. 94 %, *p* < .01), grade 3 acute GI complications (20 % vs. 56 %, *p* < .01), and grade 2 or higher chronic GI complications (3 % vs. 28 %, *p* < .01); and fewer grade 3 chronic GI complications (3 % vs. 19 %, not significant). The 3-year cumulative incidences of grade 2 or higher chronic GI complications were significantly lower with IMRT compared to 3DCRT (3 % vs. 45 %, HR = 7.5, 95 % CI = 1.2-15.0, *p* < .02) and in patients with V40 of the small bowel loops of ≤340 mL compared to those with >340 mL (3 % vs. 45 %, HR = 7.7, 95 % CI = 3.2-61.0, *p* < .001) (Fig. 3). Patients treated with IMRT had a higher incidence of grade 3 acute HT complications (38 % vs. 63 %, *p* < .05).

**Table 5:** Acute and chronic complications of 3DCRT and IMRT

			3DCRT		IMRT		
			n	(%)	n	(%)	*p*
Acute	GI	≥G2	30	94	19	63	<0.01
		G3	18	56	6	20	<0.01
	GU	≥G2	1	3	0	0	0.329
		G3	0	0	0	0	N.S.
	HT	≥G2	27	84	28	93	0.265
		≥G3	12	38	19	63	<0.05
Chronic	GI	≥G2	9	28	1	3	<0.001
		G3	6	19	1	3	0.055
	GU	≥G2	2	6	1	3	0.593
		G3	0	0	0	0	N.S.
	leg edema	≥G2	4	13	4	13	0.922
		G3	0	0	0	0	N.S.

*3DCRT* three-dimensional conformal radiation therapy, *IMRT* intensity-modulated radiation therapy, *GI* gastrointestinal, *GU* genitourinary, *HT* hematologic toxicity

**Fig. 4:**
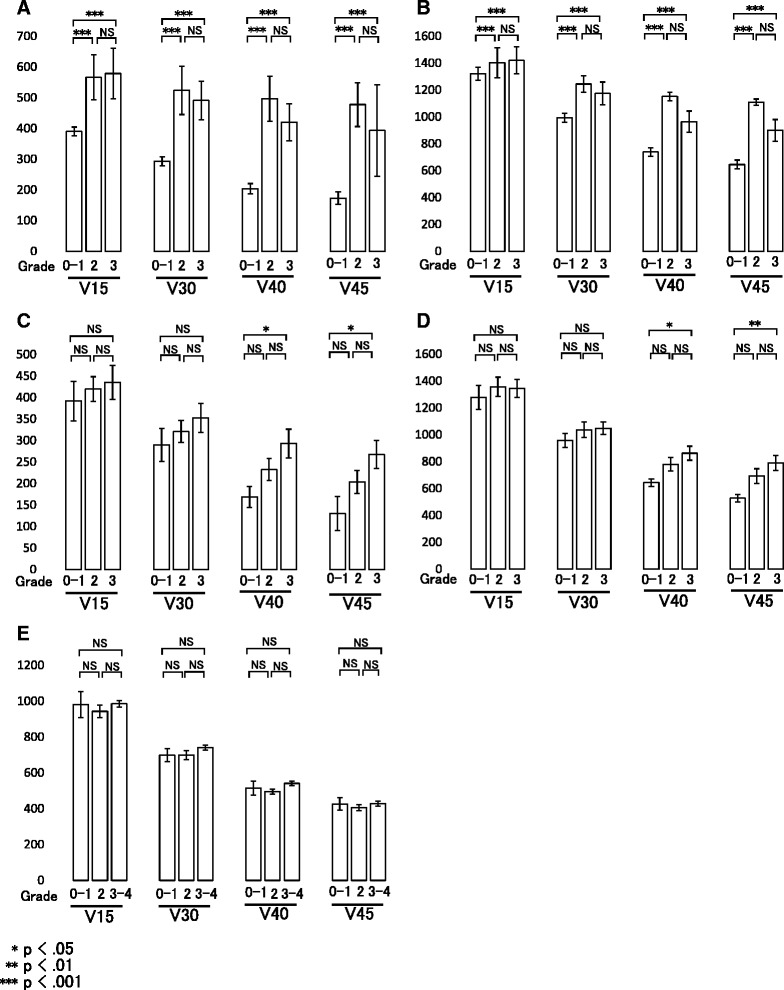
Correlations of grades of complications with DVH parameters for **a** small bowel loops for chronic GI complications, **b** bowel bag for chronic complications, **c** small bowel loops for acute GI complications, **d** bowel bag for acute GI complications, and **e** pelvic bone for acute HT complications in IMRT and 3DCRT

Correlations between grades of complications and DVH parameters (V15-45) for all patients are shown in Fig. 4. Patients with grade 2 or higher chronic GI complications had significantly greater V15-45 volumes in the small bowel loops and bowel bag (Fig. 4a, b). Patients with grade 3 acute GI complications had significantly greater V40 and V45 volumes in the small bowel loops and bowel bag, compared to patients with grade 0–1 complications (Fig. 4c, d). The grades of acute GI complications increased in a volume-dependent manner based on V40 and V45 of the small bowel loops or bowel bag, although without significance. These data indicate that V40 and V45 of small bowel loops or bowel bag were predictive for development of both acute and chronic GI complications. There was no correlation between the grades of acute HT complications and DVH values in pelvic bone (Fig. 4e).

## Discussion

This study provides a comparison of the outcomes of patients with uterine cervical cancer treated with postoperative IMRT versus postoperative 3DCRT concurrent with weekly nedaplatin. There were no significant differences in OS, LRC and PFS between the cohorts, but IMRT reduced acute and chronic GI complications compared with 3DCRT. Previous reports have suggested a potential role for IMRT in adjuvant treatment of cervical cancer with adverse risk factors [12, 13]. Our retrospective data support the benefit of IMRT in reducing GI complications in postoperative chemoradiation for cervical cancer. A randomized phase III trial of postoperative treatment of endometrial and cervical cancer (RTOG1203) is ongoing for comparison of outcomes and complications between IMRT and 3DCRT, with a focus on acute GI complications.

We used nedaplatin as concurrent chemotherapy with RT. Nedaplatin (cis-diammine-glycoplatinum), a derivative of cisplatin, was developed by Shionogi Pharmaceutical Company in Japan, with the aim of reduced renal and gastrointestinal toxicity, but similar effectiveness, compared to cisplatin [14]. Nedaplatin has a particularly favorable efficacy postoperatively [5, 15] and in locally advanced cervical cancer [16]. Therefore, we considered that substitution of nedaplatin for cisplatin in concurrent chemotherapy may be beneficial for patients with cervical cancer.

We previously reported that V15-V45 of the small bowel loops has high accuracy for prediction of chronic GI complications and that V40 of the small bowel loops >340 mL is an independent risk factor for chronic GI complications in patients treated with adjuvant whole-pelvic RT using conventional 2D or 3DCRT [4]. However, dose patterns differ considerably between conventional 2D or 3DCRT and IMRT, and this raises the question of whether our previous findings for predictors apply in IMRT. In the current study, patients with grade 2 or higher chronic GI complications had significantly greater V15-V45 of the small bowel loops and the 3-year cumulative incidences of these complications were 3 and 45 % in patients with V40 values of ≤340 mL and >340 mL, respectively (*p* < .001). Therefore, our previous findings for predictors of chronic GI complications after 2D or 3DCRT are also useful in IMRT.

Chopra et al. found that V15 of the small bowel loops and large bowel loop are independent predictors of chronic grade 3 or higher complications [17], and recommended risk cut-off values of <275 mL and <250 mL, respectively. The difference in cut-off values in Chopra et al. and the current study may be due to treatment with or without brachytherapy, different endpoints (grade 2 or 3), different chemotherapy regimens (nedaplatin or cisplatin), and the higher DVH parameters of the small bowel loops and large bowel loop in our study (Table 3). In fact, only 9 of our patients (15 %) had V15 of the small bowel loops <275 mL. Therefore, the difference in DVH parameters might be due to differences in the physical characteristics of the patients in the two studies. There were many thin patients in our study (41/62 patients had BMI <22). However, a further study is required to determine the correlation between physical characteristics and bowel volume, and to seek better predictors of chronic GI complications.

A predictive model of acute GI complications is described in the Quantitative Analyses of Normal Tissue Effects in the Clinic (QUANTEC) guidelines [18]. QUANTEC indicated that V15 of the small bowel loops should be <125 mL or V45 of the bowel bag should be <195 mL to reduce the grade 3 complication rate to <10 %. However, in the current study the mean volumes of V15 of the small bowel loops for 3DCRT and IMRT were 428 and 411 mL, respectively, and the mean volumes of V45 of the bowel bag for 3DCRT and IMRT were 891 and 489 mL, respectively (Table 3). Therefore, the volumes of the small bowel loops or bowel bag were in excess of the QUANTEC volumes to reduce grade 3 complications to <10 % in both IMRT and 3DCRT. Consequently, a high rate of grade 3 acute GI complications of 20 % occurred in the IMRT group, but this was still less than the rate of acute GI complications after 3DCRT.

We previously reported that the small bowel loops may be better predictors of chronic GI complications compared to the bowel bag in 2D and 3DCRT [4]. However, in this study using 3DCRT and IMRT, patients with grade 2 or higher chronic GI complications had significantly greater V15-V45 volumes in the small bowel loops and bowel bag (Fig. 4a, b); and patients with grade 3 acute GI complications had significantly greater V40 and V45 volumes in the small bowel loops and bowel bag (Fig. 4c, d). The grades of acute GI complications also increased in a volume-dependent manner using V40 and V45 of the small bowel loops or bowel bag, although the relationship was not significant. Wedlake et al. found that cumulative acute GI symptoms measured by questionnaire are associated with consequential late symptoms [19]. Additionally, QUANTEC predicted that chronic GI complications are likely to be related to maximum dose or volume threshold parameters that are qualitatively similar to those related to the risk of acute GI complications [18]. Collectively, these findings indicate that bowel bag parameters are useful predictors of chronic and acute GI complications in 3DCRT and IMRT.

Patients treated with IMRT, for which the pelvic bone was not used as a planning constraint, showed a greater incidence of grade 3 or higher acute HT complications (*p* < .05). Klopp et al. found that V40 of the pelvic bone predicted development of HT complications in the RTOG 0418 prospective trial [20]. Therefore, in our study, the cause of the significant increase in HT complications in IMRT may have been a significantly greater V40 of the pelvic bone, compared to patients who received 3DCRT (Fig. 2 and Table 4). These data indicate that bone marrow sparing IMRT is useful because IMRT is particularly effective at reducing the volume receiving a relatively high dose. Conversely, Mell et al. and Albuquerque et al. found that V10 and V20 of the pelvic bone more accurately predicted HT complications, compared to V30 or V40 [11, 21]. However, patients in our study who received IMRT had a greater incidence of HT complications and a significantly reduced V20 of the pelvic bone compared to patients who received 3DCRT (Table 4). These data indicate that the relationship between HT complications and DVH parameters of the pelvic bone is complicated. Therefore, future studies are required to examine the clinical benefit of IMRT in reducing HT complications and to validate the critical DVH predictors of these complications.

The findings in this study should be interpreted with an understanding of the following limitations. First, the heterogeneity in the treatment planning approach over the periods of the study (3DCRT and IMRT); the low number of events, especially in IMRT; and the lack of a pre-specified model or protocol are important limitations of the data and analysis. Second, we used weekly nedaplatin as concurrent chemotherapy, whereas chemoradiation therapy with 40 mg/m^2^ weekly cisplatin is now accepted as the standard first-line treatment. Therefore, we cannot exclude the possibility that the DVH parameter predictors found in this study may be chemotherapy-type specific, particularly as Bazan et al. showed that DVH predictors for acute HT complications in patients receiving IMRT are dependent on the type of chemotherapy [22].

## Conclusions

We conclude that IMRT is useful for decreasing GI complications without worsening outcomes. Further studies are required to identify critical DVH parameters for avoidance of acute HT complications.

## Abbreviations

IMRT: Intensity-modulated radiation therapy
3DCRT: Three-dimensional conformal radiation therapy
DVH: Dose-volume histogram
RT: Radiation therapy
OS: Overall survival rate
LRC: Loco-regional control rate
PFS: Progression-free survival rate
GI: Gastrointestinal
2D: Two-dimensional
3D: Three-dimensional
CRT: Conformal radiation therapy
CTV: Clinical target volume
RTOG: the Radiation Therapy Oncology Group
GU: Genitourinary
HT: Hematologic
CI: Confidence interval
QUANTEC: the Quantitative Analyses of Normal Tissue Effects in the Clinic
